# Negative Energy Balance in Transition Cows Induces Complex Changes in Lipid Profile of Milk

**DOI:** 10.3390/metabo16020103

**Published:** 2026-01-30

**Authors:** Zhiqian Liu, Vilnis Ezernieks, Joanne E. Hemsworth, Coralie M. Reich, Carolyn R. Bath, Monique J. Berkhout, Muhammad S. Tahir, Leah C. Marett, Amanda J. Chamberlain, Mike E. Goddard, Ruidong Xiang, Simone J. Rochfort

**Affiliations:** 1 Agriculture Victoria Research, AgriBio, 5 Ring Road, Melbourne, VIC 3083, Australia; vilnis.ezernieks@agriculture.vic.gov.au (V.E.); joanne.hemsworth@agriculture.vic.gov.au (J.E.H.); coralie.reich@agriculture.vic.gov.au (C.M.R.); carolyn.bath@agriculture.vic.gov.au (C.R.B.); sajid.tahir@agriculture.vic.gov.au (M.S.T.); amanda.chamberlain@agriculture.vic.gov.au (A.J.C.); michael.goddard@agriculture.vic.gov.au (M.E.G.); ruidong.xiang@agriculture.vic.gov.au (R.X.); simone.rochfort@agriculture.vic.gov.au (S.J.R.); 2 Agriculture Victoria Research, Ellinbank Centre, Ellinbank, VIC 3821, Australia; monique.berkhout@agriculture.vic.gov.au (M.J.B.); leah.marett@agriculture.vic.gov.au (L.C.M.); 3 School of Agriculture and Food, Faculty of Veterinary and Agricultural Sciences, The University of Melbourne, Melbourne, VIC 3010, Australia; 4 School of Applied Systems Biology, La Trobe University, Melbourne, VIC 3083, Australia

**Keywords:** cows, milk, lipidomic profiling, biomarkers, negative energy balance

## Abstract

Background: Negative energy balance (NEB) during the transition period is associated with profound changes in the body condition and metabolic dynamics of dairy cows. However, the detailed lipidomic changes in milk induced by NEB are unclear, and lipid biomarkers that indicate the energy status of cows remain to be established. Methods: Using a combination of GC-FID, HILIC-MS and RP-LC-MS, we performed a systematic comparison of lipid composition between early lactating (DIM: 5–14) and mid-lactating (DIM: 65–80) milk. Results: We found that NEB in cows caused a profound modification in the profile of all the lipid classes surveyed, including phosphatidylcholine (PC), phosphatidylethanolamine (PE), phosphatidylserine (PS), phosphatidylinositol (PI), sphingomyelin (SM), lysophosphatidylcholine (LPC), PC-plasmalogen (PCP), PE-plasmalogen (PEP), lactosylceramide (LacCer), acylcarnitine (AcylCar) and triglycerides (TAGs). Except for LPC and AcylCar, which were reduced and increased, respectively, by NEB, the responses of other lipid classes varied across different species. For phospholipids and TAGs, species containing de novo FAs (C4:0–C16:0) and odd-chain FAs (C15:0 and C17:0) were markedly downregulated, whereas those comprising long-chain preformed FAs were upregulated by NEB. Conclusions: Comprehensive lipidomic profiling of early and mid-lactating milk from two large cohorts of cows allowed us to identify nine lipids (PE 33:1, LacCer 32:1, LacCer 39:1, LacCer 41:1, SM 36:1, SM 36:2, SM 37:1, PEP 38:4 and PEP 38:5) as potential biomarkers of NEB in dairy cows.

## 1. Introduction

In high-producing dairy cows, the onset of lactation brings about a dramatic increase in energy demand, often surpassing the energy intake from feed. This results in a metabolic condition known as negative energy balance (NEB), which typically occurs during the early postpartum period [1]. To compensate for the energy deficit, cows mobilize body fat reserves, leading to elevated levels of circulating non-esterified fatty acids (NEFAs) and ketone bodies such as β-hydroxybutyrate (BHBA) in the bloodstream [2]. Early detection of severe NEB enables intervention to help mitigate its negative effects, which include impaired reproductive performance, increased disease susceptibility and reduced milk yield [3,4]. Currently, the body condition score, milk fat-to-protein ratio, and blood NEFA and BHBA levels are used as indicators for NEB in dairy cows [5,6], each having pros and cons.

The circulating NEFAs are taken up by the mammary gland and incorporated into milk fat, leading to an altered milk lipid profile in transition cows. Since milk fatty acids (FAs) can be determined readily by mid-infrared spectroscopy (MIR), numerous studies have been conducted to explore the correlation between the milk FAs and energy status of cows [7,8,9]. Several FAs (especially C14:0, C18:0 and C18:1) and the FA ratio (e.g., C18:1/C15:0) have been reported as promising biomarkers for diagnosing NEB [10,11,12,13,14].

Milk FAs are a complex mixture, comprising de novo FAs (C4:0 to C16:0) synthesized in the mammary gland, preformed FAs (e.g., C18:0, C18:1, C18:2 and C18:3) derived from the digestion of plant lipids, NEFAs generated from adipose tissue, and odd- and branched-chain FAs (mainly C15:0 and C17:0) produced by rumen bacteria. Moreover, the milk FA profile is highly dynamic and both de novo and preformed FAs can be influenced by various factors like the stage of lactation [15,16,17], the diet of the animal [5,18], the genetic background of the animal [19,20] and the health status of cows [21]. The confounding effects between all these factors are not fully understood. Consequently, the prospect of using milk FAs as reliable biomarkers for predicting NEB in dairy cows remains questionable.

Apart from triglycerides (TAGs), which are a direct reflection of FA supply in the mammary gland, bovine milk contains hundreds of polar lipid species [22]. Polar lipids, which contain one or two FA moieties, are not only the major components of the milk fat globule membrane, but they are also bioactive molecules involved in cell signalling, stress responses, immune reaction and brain development [23,24]. Since NEB in cows is a condition closely associated with aberrant lipid metabolism, we hypothesized that the polar lipid signature of milk would be profoundly altered by NEB in transition cows and some polar lipid molecules in milk might be potential biomarkers for diagnosing NEB in dairy cows.

To test this hypothesis, we undertook a systematic comparison of the major lipid species of pooled milk samples from dairy cows at 1–2 weeks and 9–12 weeks post-calving, representing, respectively, the status of NEB and positive energy balance (PEB, i.e., energy intake exceeding demands). In addition, by performing chemometric analysis of data obtained from targeted lipidomic profiling of individual milk samples from two large cohorts over two years, reproducible lipid markers associated with NEB in cows were identified.

## 2. Materials and Methods

### 2.1. Milk Sample Collection

This research was conducted at the Agriculture Victoria Research Ellinbank SmartFarm in Victoria, Australia (38°14′ S, 145°56′ E), with approval from the Department of Energy, Environment and Climate Action (DEECA) Agricultural Research and Extension Animal Ethics Committee (approval code: AEC 2022-04; approval date: 11 May 2022). All procedures were conducted in accordance with the Australian Code of Practice for the Care and Use of Animals for Scientific Purposes (NHMRC, 2013).

Cows were grazing pasture and supplemented twice daily with a grain mix determined by general farm practice throughout lactation. Within a year, all cows sampled were offered the same diet. The pasture on offer was predominantly perennial ryegrass and was allocated to cows at approximately 25 kg DM/cow per day. The grain mix was offered to individual cows at milking times in the dairy parlour; generally comprised wheat, barley and canola meal; and was offered at amounts ranging from 6 to 8 kg/cow/day, depending on the stage of lactation and pasture availability, and it was formulated to meet the cow’s nutritional requirements using CPM Dairy version 3.0.8 (Cornell–Penn–Miner Institute, The University of Pennsylvania, Philadelphia, PA, USA). The cows were milked twice daily at ~7:00 and 15:00.

Milk samples (about 50 mL) were collected during morning milking from a cohort of 149 and 225 spring-calving (July–September) Holstein-Friesian cows in 2022 and 2023, respectively, at two lactation stages—early lactation (5–14 DIM), and mid-lactation and non-pregnant (65–80 DIM)—representing, respectively, the status of NEB and PEB. Each cohort comprised both primiparous heifers and multiparous cows (age: 2–11 years; parity: 1–9; average daily milk production across the lactation: 33 L). The majority (>75%) of cows were free of metabolic disease events throughout the experiment period, and the prevalence of subclinical ketosis (as judged by blood BHBA > 1.4 mmol/L) was <10%. Cows that were sampled in 2022 were not sampled again in 2023. The large and heterogenous cohorts aimed to cover the inter-cow variation in response to NEB. Milk samples were transported on ice to the laboratory and stored at −80 °C.

### 2.2. Chemicals

Mouse Splash^®^ Lipidomix standards (a mix of 14 deuterated standards) and lactosylceramide (LacCer) standards were purchased from Avanti Lipids (Alabaster, AL, USA). The acylcarnitine (AcylCar) standards were from Sigma-Aldrich (St. Louis, MO, USA). The solvents used for milk lipid extraction and mobile phase preparation were of HPLC or LC-MS grade. The methanol and isopropanol were from Fisher Chemical (Pittsburgh, PA, USA), chloroform from Sigma-Aldrich, acetonitrile and butanol from Merck (Darmstadt, Germany), and acetonitrile containing 0.1% formic acid from Fisher Chemical. The ammonium formate (a mobile phase additive) was of analytical grade (Sigma-Aldrich). 

### 2.3. Sample Preparation for LC-MS and GC Analysis

To reveal the influence of NEB on milk FA and lipid composition, pooled milk samples (from 15 random cows of the 2022 cohort) from the two time points were compared. FA composition was determined by GC-FID (Agilent Technologies, Santa Clara, CA, USA) according to the direct methylation method [25]. Lipidomic profiling was carried out by LC-MS following the one-phase extraction procedure [26]; the labelled Lipidomix standards were added to the samples prior to extraction (as internal standards, ISs).

For the identification of lipid biomarkers associated with NEB in cows, 143 (from the 2022 cohort) and 218 (from the 2023 cohort) individual milk samples from the two time points were extracted by the one-phase method prior to LC-MS analysis.

### 2.4. Quantification of Milk Lipids

HILIC-MS was employed for the quantification of polar lipids in pooled samples. The LC separation was achieved on a Vanquish UHPLC system (Thermo Fisher Scientific, Waltham, MA, USA) with a HILIC column (150 × 4.6 mm, 2.6 µm, Phenomenex, Torrance, CA, USA) maintained at 30 °C. The mobile phase was composed of 10 mM ammonium formate (A) and acetonitrile containing 0.1% formic acid (B). The elution was performed by a linear increase in mobile phase A from 2 to 40% over 16 min with a flowrate of 0.5 mL/min.

For the TAG quantification of pooled samples, RP-LC-MS was utilized. The LC separation was performed on the same Vanquish UHPLC system with a C8 column (150 × 4.6 mm, 2.6 µm, Agilent Technologies) maintained at 50 °C. The mobile phase was composed of water/acetonitrile (40:60, *v*/*v*) containing 10 mM ammonium formate (A) and acetonitrile/isopropanol (10:90, *v*/*v*) containing 10 mM ammonium formate (B). The elution was performed by a linear increase in mobile phase B from 32 to 97% over 21 min with a flowrate of 0.25 mL/min.

A Q Exactive Plus mass spectrometer (Thermo Fisher Scientific) equipped with a heated electrospray ionization (HESI) source was used for the detection of all lipids. The heated capillary was maintained at 300 °C with a source heater temperature of 300 °C, and the sheath, auxiliary and sweep gases were at 30, 10 and 0 units, respectively. The instrument was operated in both positive (4.2 kV) and negative (3.6 kV) full scan mode (150–1600 *m*/*z*) at a resolution of 70,000. The concentration of PC, PE, PS, SM, PI, LPC, PCP, PEP and TAG in pooled samples was calculated using the one-point calibration method—i.e., concentration of a lipid species = (peak area of the lipid species/peak area of the IS) × concentration of the IS—whereas that of LacCer and AcylCar was determined by an external calibration method.

To identify lipid biomarkers for NEB in cows, all individual samples of the 2022 and 2023 cohorts were analyzed in a randomized order using a higher-throughput HILIC-MS workflow as described previously [27]. The MS response fluctuation was monitored by analyzing the Lipidomix standard mix and pooled QC sample after every 20 samples. The relative abundance (peak area) of lipids was used in chemometric analysis and predictive modelling.

### 2.5. Data Analysis

Statistical comparison of the lipid concentrations in pooled milk samples from cows in NEB and PEB was conducted by Student’s *t*-tests. Chemometric analysis of the lipidomic data obtained from the whole cohort of 2022 and 2023 was carried out with MetaboAnalyst 6.0 (http://www.metaboanalyst.ca) [28]. Predictive models of the energy status of dairy cows based on all lipidomic data were built with MATLAB 2024a (MathWorks) and PLS-Toolbox 9.3.1 (Eigenvector). An initial model was built with the 2022 data matrix split into calibration and validation datasets using the Kennard–Stone algorithm with Euclidean distance measures, retaining 66% of the data for calibration. Both X and Y blocks were autoscaled. Cross-validation used the Venetian blinds method with 10 splits and blind thickness = 1. The resultant model was saved and tested for its predictiveness on the 2023 dataset. The resultant model was also checked for robustness by permutation testing (*n* = 200).

## 3. Results

### 3.1. Effect of NEB on Milk FA Composition

The FA composition of milk, as judged by the % in total fat, varied greatly between the two energy statuses of cows. The % of medium-chain de novo FAs C10:0 to C16:0, and most of the odd- and branched-chain FAs (C15:0, C15:0iso, C15:0anteiso, C17:0iso and C17:0anteiso) in milk was significantly lower, whereas that of preformed FAs C18:0, C18:1, C18:2, C18:3, C20:4n6, C20:5, C22:5 and C22:6 was significantly higher when cows were in NEB compared to PEB. Short-chain de novo FAs C4:0-C8:0 did not follow the same pattern as medium-chain de novo FAs in relation to the energy status of cows (Table 1). The % of total saturated FAs increased slightly from NEB to PEB in cows (Table 1).

### 3.2. Effect of NEB on Milk Lipid Composition

By combining HILIC-MS and RP-LC-MS, 200 polar lipids and 81 TAGs were identified and quantified from the pooled milk samples; the full list of lipids and their concentrations is given in Table S1. Overall, >85% of polar lipids and >90 of TAGs displayed a significant difference (*p* < 0.05) between NEB and PEB in cows. The lipidomic change associated with the energy status of cows varied with both lipid classes and lipid species within the same class.

**Figure 1:**
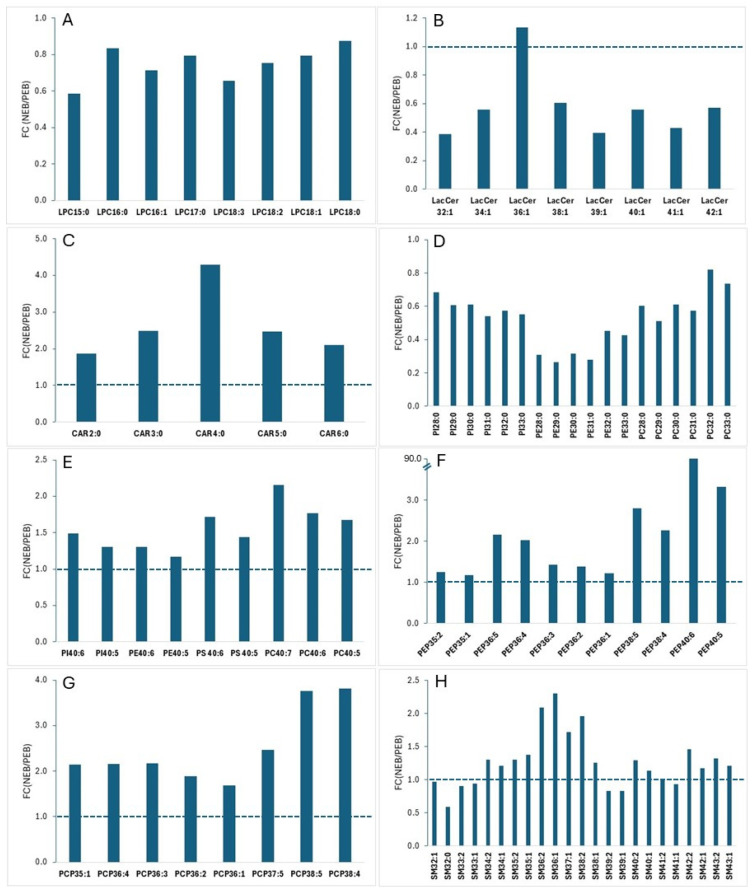
Comparison of milk polar lipid abundance from cows in NEB and PEB. (**A**) LPC; (**B**) lactosylceramide (LacCer); (**C**) acylcarnitine (AcylCar); (**D**) PE-plasmalogen (PEP); (**E**) PC-plasmalogen (PCP); (**F**) PI, PE and PC species with total acyl carbon < 34; (**G**) PI, PE, PS and PC species with a total acyl carbon of 40 and double bonds > 4. (**H**) SM class. For all plots, values are the means of 4 measurements of pooled samples. FC (fold change) = NEB/PEB.

All the LPC species and the majority of the LacCer species (except LacCer 36:1) were found at a lower abundance when cows were in NEB, with a fold change (FC) (NEB/PEB) ranging from 0.58 to 0.88 for LPC and from 0.38 to 0.60 for LacCer (Figure 1A,B). By contrast, an accumulation of all Acylcar species was detected when cows were in NEB (FC between 1.88 and 4.29) (Figure 1C).

For the most abundant polar lipid classes of milk PC, PE, PI and PS, the response to NEB was multifaceted depending on the FA structure of each species. For example, all PI, PE and PC species having a total acyl chain length < 34 and nil double bond recorded a marked reduction under the NEB condition (FC between 0.27 and 0.82) (Figure 1D). On the other hand, PI, PE, PS and PC molecules with a total acyl chain length of 40 and double bond ≥ 5, including PI 40:6/40:5, PE 40:6/40:5, PS 40:6/40:5 and PC 40:7/40:6/40:5, followed an opposite trend, i.e., a marked increase in early lactating milk when cows were in NEB (FC: 1.17–2.15; Figure 1E). The pattern of PC- and PE-plasmalogens differed from PC and PE. While PEP and PCP species with a total acyl length < 34 exhibited a smaller fluctuation (Table S1), a significant enrichment of those having a total acyl chain length > 34 was observed when cows were in NEB (Figure 1F,G).

As for SM, a class of sphingolipids, no clear rule can be drawn between the energy status of cows and the fatty acyl structure of individual species. Several medium-chain species including SM 36:1, SM 36:2, SM 37:1 and SM 38:2 appeared to be markedly boosted by the NEB condition of cows (Figure 1H).

**Figure 2:**
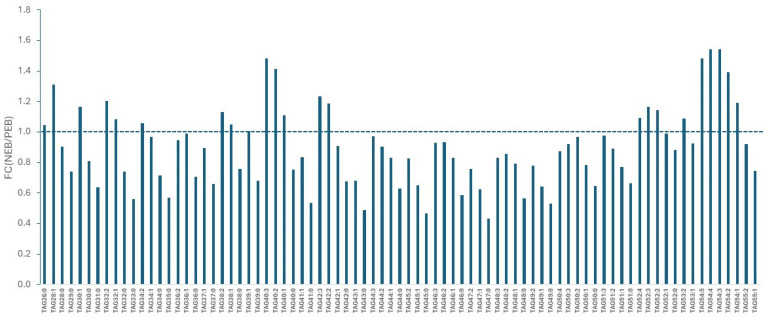
Comparison of milk TAG abundance from cows in NEB and PEB. Values are the means of 4 measurements of pooled samples. FC (fold change) = NEB/PEB.

In the case of TAGs, a variable response to the NEB condition of cows was again detected: a significant decrease in saturated species, especially those with an odd total acyl carbon number, such as TAG 43:0, TAG 45:0 and TAG 47:0, and a significant enrichment of unsaturated species, such as TAG 40:3, TAG 54:5, 54:4 and 54:3 (FC > 1.4) (Figure 2).

### 3.3. Lipid Biomarker Detection for NEB in Cows

**Figure 3:**
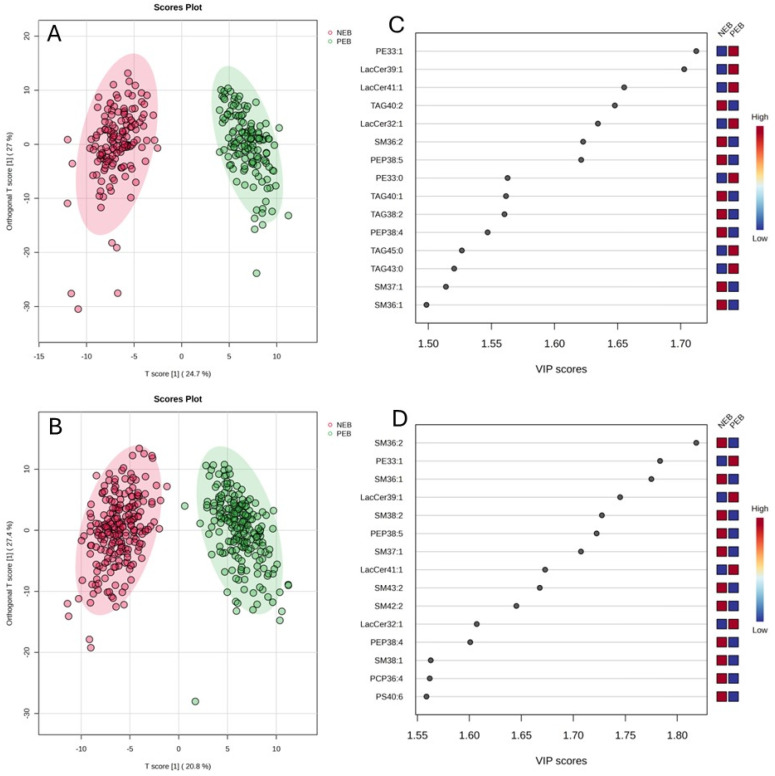
OPLS-DA plot of milk samples collected from cows in NEB and PEB in 2022 (*n* = 143) (**A**) and 2023 (*n* = 218) (**B**), and the viable importance in projection (VIP) scores of the top 15 features differentiating the energy status of cows for 2022 (**C**) and 2023 (**D**) cohorts.

Milk samples from two large cohorts of cows were analyzed to identify biomarkers for NEB in cows. A total of 202 and 201 polar lipid and TAG species were surveyed for all the individual samples of the 2022 and 2023 cohorts, respectively. For the 2022 cohort, 153 out of the 202 lipid features were found to be significantly different (*p* < 0.00025, Bonferroni adjusted *p* value) between cows in NEB and PEB. The number of lipids showing a significant difference increased slightly to 156 (*p* < 0.00025) for the 2023 cohort. When the whole data matrix of each cohort was subjected to orthogonal partial least square discriminant analysis (OPLS-DA), a clear separation of milk samples by energy status of cows was observed for both cohorts (Figure 3A,B), indicating that milk lipid composition is influenced by the energy status of dairy cows.

The top 15 most differential lipid species identified by the OPLS-DA model for the 2022 cohort and 2023 cohort are shown in Figure 3C and Figure 3D, respectively. Each list contains both up- and downregulated features with a VIP score > 1.5, indicating their significant role in discriminating the two groups. An inconsistency between the two cohorts was discerned among the top 15 lipids. For example, 5 TAGs (TAG 40:2, TAG 40:1, TAG 38:2, TAG 45:0 and TAG 43:0) were identified from the 2022 cohort, but none from the 2023 cohort. Conversely, 7 SM species (SM 36:1, SM 36:2, SM37:1, SM 38:1, SM38:2, SM 42:2 and SM 43:2) were detected from the 2023 cohort, but only 3 from the 2022 cohort. Despite the discrepancy, out of the top 15 lipids identified independently from each cohort, 9 (PE 33:1, LacCer 32:1, LacCer 39:1, LacCer 41:1, SM 36:1, SM 36:2, SM 37:1, PEP 38:4 and PEP 38:5) were common across the two cohorts.

**Figure 4:**
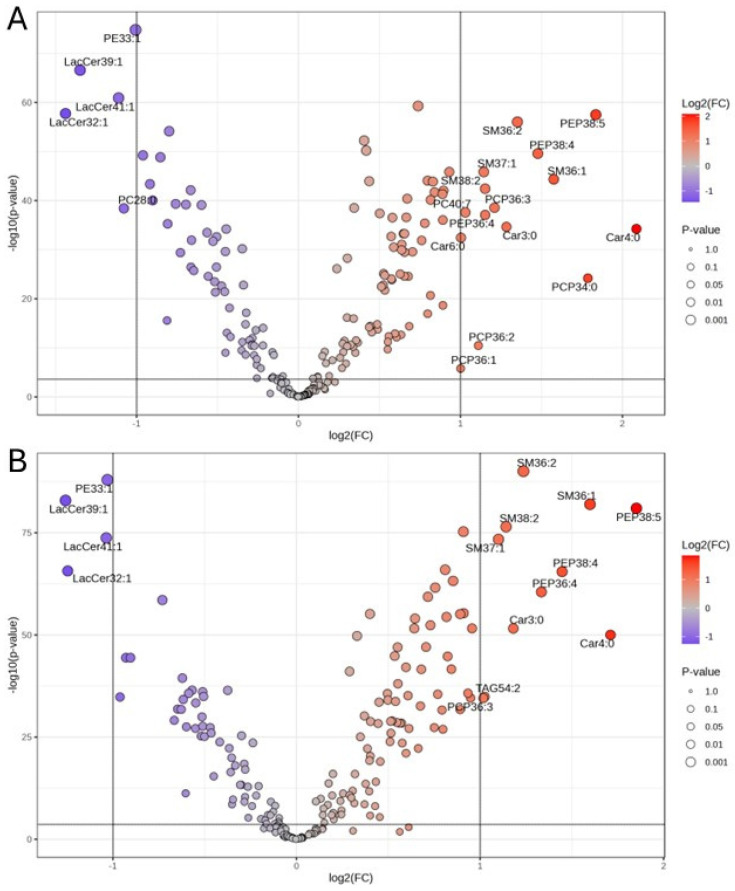
Volcano plot of upregulated (in red) and downregulated (in blue) lipid species (NEB vs. PEB) detected from 2022 cohort (**A**) and 2023 cohort (**B**). Only lipids with *p* < 0.00025 and FC > 2.0 are labelled.

Taking into consideration both the *p* value (*p* < 0.00025) and FC (>2 or log_2_ FC > 1), the volcano plot allowed the identification of 15 and 11 upregulated and 5 and 4 downregulated lipids (NEB vs. PEB) from the 2022 and 2023 cohorts, respectively (Figure 4A,B). The upregulated lipids were mostly from the Acylcar, SM, PEP and PCP families, whereas the downregulated ones were mainly LacCer molecules apart from a single PE species. Four downregulated lipids (PE 33:1, LacCer 32:1, LacCer 39:1 and LacCer 41:1) and 10 upregulated lipids (Car 3:0, Car 4:0, SM 36:1, SM 36:2, SM 37:1 and SM 38:2, PEP 38:5, PEP 38:4, PEP 36:4 and PCP 36:3) were common across the two cohorts.

When the results from the OPLS-DA and the volcano plot for both the 2022 and 2023 cohorts were combined, nine lipids (PE 33:1, LacCer 32:1, LacCer 39:1, LacCer 41:1, SM 36:1, SM 36:2, SM 37:1, PEP 38:4 and PEP 38:5) consistently stood out as the most promising biomarkers for detecting NEB in dairy cows.

### 3.4. Prediction of Energy Status Based on Milk Lipidomic Data

**Figure 5:**
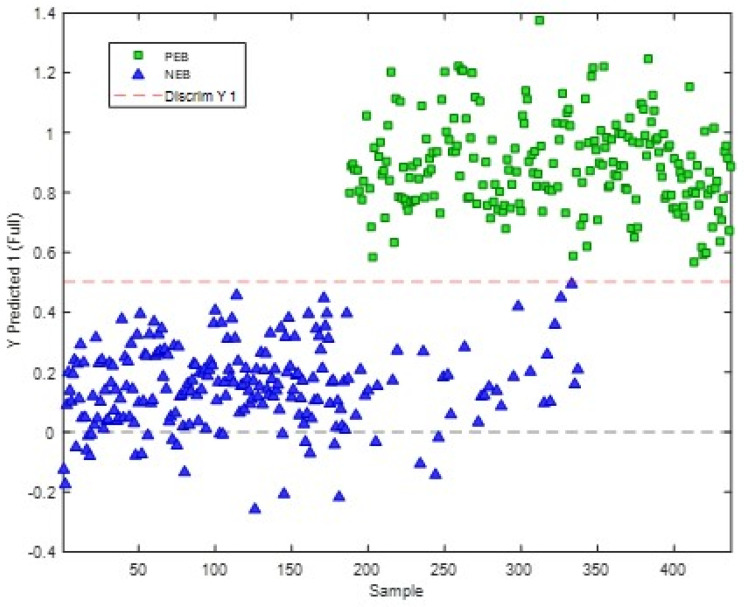
Prediction plot for samples collected in 2023 using PLS-DA model built with the 2022 lipidomic data.

The 2022 data was used to build a model for the prediction of the energy status of cows by splitting the data into calibration and validation datasets using the Kennard–Stone algorithm with Euclidean distance measures, retaining 66% of the data for calibration, with the remaining data used to test the model. The PLS-DA model gave calibration, cross-validation and prediction errors of 0%, with sensitivity and specificity of 1 (four latent variables, capturing 65.48% of the X block data). This model was saved, and the 2023 X and Y data were loaded for validation. The model allowed the accurate prediction of all the samples (Figure 5). The error of prediction was 0%, with sensitivity and specificity of 1. Permutation testing (*n* = 200) suggested that the model was not overfitted (*p* < 0.005).

## 4. Discussion

Given the complexity in determining the energy status of cows in a pasture-based system, all cows at 5–14 DIM were considered to be in NEB and those at 65–80 DIM in PEB conditions. This assumption was supported by the literature. For example, 75% of cows in the UK were in NEB during the first 20 DIM [29]. Our comprehensive biochemical analysis data (blood NEFA, BHBA, glucose, cholesterol and so on) of the cows, which will be published elsewhere, also supported the energy status classification. For example, 91% of the 2022 cows had a blood NEFA level ≥ 0.4 mmol/L (mean: 0.58 ± 0.20) and/or a BHBA level ≥ 0.8 mmol/L at 5–14 DIM, whereas at 65–80 DIM, only 5% of the same cohort had a blood NEFA level ≥ 0.4 mmol/L (mean: 0.22 ± 0.11). Clearly, our group assignment of the energy status of cows based purely on DIM may lead to misplacement of a small number of animals, which is a limitation of this study. It is worth mentioning that the second time point (65–80 DIM) was chosen to consider the 12-month calving interval and at the same time to ensure that the cows were not pregnant, so that pregnancy would not be impacting energy demands.

A systematic comparison of the milk FA profile, polar lipids and TAGs between cows in NEB and PEB was one of the objectives of this study. To achieve this, pooled samples from 15 random cows (representing around 10% of the cohort) were analyzed, which were deemed adequate to represent a population while allowing a range of analytical tools (GC-FID, HILIC-MS and RP-LC-MS) to be applied to a smaller number of samples.

The composition of milk FAs undergoes dynamic changes throughout the lactation cycle in dairy cows, reflecting shifts in metabolic and physiological adaptations. It is well established that, under NEB conditions, there is an increased incorporation of long-chain FAs (primarily derived from mobilized adipose tissue) into milk fat [30,31]. This shift is often accompanied by a decrease in the de novo synthesis of short- and medium-chain FAs in the mammary gland [32]. Our results are in partial agreement with the literature regarding FA composition change in the milk of transition cows. While we observed a reduction in the proportion of some de novo FAs (C10:0–C16:0) and a concomitant increase in the proportion of long-chain preformed FAs, other de novo FAs (including C4:0, C6:0 and C8:0) did not decrease when cows were in NEB. This may be because these short-chain FAs are also precursors of de novo FA synthesis, so a higher % of these FAs may simply be a result of reduced elongase activity to transform them into longer chain FAs in transition cows. It is interesting to note that C15:0 and branched-C15:0, also considered as preformed FAs and often overlooked in previous studies, displayed a pattern resembling that of de novo FAs (e.g., C14:0). It is known that a small proportion of C15:0/C17:0 can be synthesized in the mammary gland from C3:0 using the de novo pathway; however, most of these FAs originate from rumen bacteria [33]. The lower level of C15:0 when cows were in NEB is thus most likely due to reduced dry matter intake and/or weakened bacterial activity in the rumen. The shift in the FA composition of milk with the energy status of cows has implications for human nutrition. Milk from cows in NEB contains a high proportion of unsaturated FAs, including ω-3 FAs, which are known to be beneficial for human health. On the other hand, milk from cows in PEB is richer in saturated FAs including branched- and odd-chain saturated FAs, which have health-protective effects against certain diseases [33]. It is worth noting that the prevalence rate of subclinical ketosis in our experimental herd was low (<10%), but that of subacute ruminal acidosis (SARA) was not determined. SARA is known to alter milk FA profiles, especially odd- and branch-chain FAs and trans FAs [34]. Consequently, the contribution of SARA to the overall results of this study could not be estimated.

While significant differences were found with most of the lipid classes surveyed, the most striking changes in lipid profile associated with NEB in cows were observed with LPC, AcylCar, PEP, PCP and LacCer. AcylCar lipids are generally not included in milk lipidomic analysis, but a few recent studies have unveiled their active role in lipid metabolism in dairy cows [27,35]. The accumulation of AcylCar in the milk of cows in NEB may result from direct transport from blood to the mammary gland, as a higher level of AcylCar was previously observed in the serum of transition cows [27]. PEP and PCP are both minor components of milk phospholipids and were largely neglected in earlier reports. Our present study revealed a widespread accumulation of PEP and PCP in cows under NEB conditions. Plasmalogens are antioxidant molecules [36,37], so the rise in PEP and PCP during the transition period may be an adaptive response to metabolic stress. LacCer lipids, a class of glycosphingolipids, were found to be substantially reduced when cows were in NEB. Apart from being a component of the fat globule membrane, the functions of these lipids in relation to the health of dairy cows are unclear.

For the major phospholipid classes, variable responses across different species to NEB appeared to be linked to their FA composition. For example, PI, PE and PC species with a total acyl carbon < 34 are mostly composed of de novo FAs and odd-chain FAs C15:0, so they displayed a significant reduction when cows were in NEB. At the same time, PI, PE and PC species containing a sum acyl structure of 40:7, 40:6 and 40:5 were all markedly increased, because these lipid molecules contained two preformed FAs. SM, one of the major polar lipid classes of milk, is known to play a role in cell signalling and apoptosis [36], but in this study, no clear fatty acyl-related pattern can be identified regarding their response to NEB, despite some species showing a great sensitivity to the energy status of cows.

In the case of TAGs, generally considered as an inert energy reserve, the response to NEB is generally in conformity with their FA structure. For example, TAG 43:0, TAG 45:0 and TAG 47:0 each contain 2–3 de novo/odd-chain FAs, with the most abundant isomers being C15:0–C12:0–C16:0, C15:0–C14:0–C:16:0 and C15:0–C16:0–C16:0/C15:0–C14:0–C18:0, respectively; as expected, they were found at a lower level when cows were in NEB. On the contrary, TAG 54:2, TAG 54:3, TAG 54:4 and TAG 54:5, each containing 3 preformed FAs (by various combinations of C18:0, C18:1 and C18:2), were detected at a higher abundance while cows were in NEB.

The pooled samples provided a detailed account of lipidomic changes in relation to the energy status of cows but cannot reveal the variation within the cohort nor the most promising biomarkers for NEB. Milk samples from the entire cohorts were thus analyzed using a higher-throughput LC-MS workflow, and the entire data matrix was subjected to chemometric analysis. Using all the lipid data, the resultant PLS-DA model showed excellent predictive power for the energy status of cows from different years/cohorts, confirming that the overall pattern in milk lipid profile induced by the NEB condition is temporally reproducible. The identification of potential lipid biomarkers was further conducted using OPLS-DA and a volcano plot.

OPLS-DA enabled the detection of lipid features that best differentiate between the NEB and the PEB groups based on VIP scores, whereas the volcano plot allowed us to pinpoint those matching both the FC and *p* value threshold. In this study, we observed a marked discrepancy in the top lipid features identified by OPLS-DA across the two cohorts/years, which may result from the year-to-year variation in environmental conditions and pasture composition. By contrast, lipid markers selected with the volcano plot are largely consistent over the two years. By combining these two complementary approaches, we were able to identify several lipids as potential biomarkers for NEB in dairy cows. Given the complexity in determining the energy status of cows using standard protocols, these lipid biomarkers may provide an alternative and non-invasive tool for the early diagnosis of cows in severe NEB, enabling early intervention to mitigate the negative impact on production and profitability. However, whether the lipid biomarkers identified and the predictive model discovered in this study can be applied to other regions or countries with different production regimes (from pure pasture-based, through to total mixed rations) and varying environmental conditions remains to be validated.

## 5. Conclusions

This study focused on the comparison of detailed lipid signatures between cows in NEB and PEB. Targeted lipidomic analysis revealed a widespread and complex pattern of milk lipid shift in response to NEB conditions. While some of the changes associated with NEB appear to be related to the FA supply in the mammary gland, the drastic up- or downregulation of LPC, LacCer, AcylCar and SM molecules induced by NEB suggests that these lipids are actively involved in the maintenance and adaptation of dairy cows during the transition period. Large cohort lipidomic profiling combined with chemometric analysis not only revealed that the energy status of dairy cows can be reliably predicted by milk lipidomic profiles, but also allowed us to identify several potential lipid biomarkers (PE 33:1, LacCer 32:1, LacCer 39:1, LacCer 41:1, SM 36:1, SM 36:2, SM 37:1, PEP 38:4 and PEP 38:5) for NEB in dairy cows. Our findings contribute to a better understanding of milk lipid dynamics in relation to the energy status of cows. Future research will focus on the validation of the promising biomarkers in multiple herds with different production systems.

## Figures and Tables

**Table 1:** Fatty acid composition (% in total fat) of milk from cows in NEB and PEB.

FA	NEB	PEB	Fold Change (NEB/PEB)	*p* Value
C4:0	2.72	1.90	1.43	0.0009
C6:0	1.94	1.74	1.11	0.0289
C8:0	1.62	1.47	1.10	0.0045
C10:0	3.62	3.74	0.97	0.0026
C12:0	3.93	4.24	0.93	0.0000
C14:0	10.93	12.58	0.87	0.0000
C15:0iso	0.20	0.26	0.77	0.0002
C15:0anteiso	0.38	0.57	0.67	0.0000
C15:0	1.16	1.61	0.72	0.0000
C16:0iso	0.22	0.27	0.81	0.0016
C16:0	28.76	31.36	0.92	0.0000
C17:0iso	0.48	0.52	0.92	0.0015
C17:0ai	0.35	0.39	0.90	0.0069
C17:0	0.82	0.79	1.04	0.0289
C18:0	13.55	12.02	1.13	0.0000
C18:1t11	2.79	2.35	1.19	0.0000
C18:1c9	19.96	18.25	1.09	0.0062
C18:2c	2.33	1.82	1.28	0.0000
C18:3n3	1.00	0.68	1.47	0.0000
C20:4n6	0.15	0.09	1.67	0.0000
C20:5n3	0.10	0.07	1.43	0.0000
C22:5n3	0.15	0.12	1.25	0.0000
C22:6n3	0.03	0.01	3.00	0.0000

NEB: negative energy balance; PEB: positive energy balance; values in the table are means of 4 measurements of pooled milk samples from 15 random cows.

## Data Availability

The original contributions presented in this study are included in the article/Supplementary Materials. Further inquiries can be directed to the corresponding author.

## Supplementary Materials

The following supporting information can be downloaded at: https://www.mdpi.com/article/10.3390/metabo16020103/s1. Table S1: Comparison of milk lipid concentration (µg/mL) between cows in NEB and PEB.
