# Supplementary material for: Negative Energy Balance in Transition Cows Induces Complex Changes in Lipid Profile of Milk
Source: Metabolites. 2026 Jan 30;16(2):103. doi: 10.3390/metabo16020103 (PMC12943591; doi:10.3390/metabo16020103)
Supplement: Supplementary file 1 [file metabolites-16-00103-s001.zip › metabolites-4081320-supplementary.pdf]

Table S-1: Comparison of milk lipid concentration (µg/mL) between cows in NEB and PEB

**Class**		**NEB**		**PEB**		**FC**	**p value**
		**Mean**	**SD**	**Mean**	**SD**		
PI	PI28:0	0.035	0.002	0.051	0.001	0.68	0.00001
	PI29:0	0.006	0.001	0.010	0.000	0.61	0.00001
	PI30:0	0.121	0.004	0.198	0.005	0.61	0.00000
	PI31:0	0.034	0.002	0.063	0.004	0.54	0.00001
	PI32:2	0.037	0.002	0.043	0.002	0.85	0.00132
	PI32:1	0.267	0.012	0.354	0.011	0.75	0.00004
	PI32:0	0.304	0.012	0.529	0.012	0.57	0.00000
	PI33:2	0.018	0.002	0.023	0.001	0.81	0.00523
	PI33:1	0.117	0.005	0.166	0.005	0.71	0.00001
	PI33:0	0.098	0.006	0.177	0.010	0.55	0.00001
	PI34:3	0.064	0.003	0.057	0.004	1.13	0.02371
	PI34:2	0.498	0.021	0.470	0.027	1.06	0.15171
	PI34:1	1.644	0.048	1.807	0.065	0.91	0.00699
	PI34:0	0.369	0.018	0.548	0.021	0.67	0.00001
	PI35:2	0.105	0.007	0.098	0.008	1.07	0.24345
	PI35:1	0.245	0.009	0.267	0.013	0.92	0.03403
	PI35:0	0.068	0.006	0.099	0.006	0.69	0.00029
	PI36:4	0.263	0.013	0.203	0.006	1.29	0.00014
	PI36:3	1.280	0.046	1.186	0.057	1.08	0.04323
	PI36:2	4.563	0.154	4.562	0.179	1.00	0.99280
	PI36:1	4.967	0.174	5.664	0.196	0.88	0.00181
	PI36:0	0.655	0.064	0.672	0.154	0.97	0.84566
	PI38:6	0.151	0.004	0.101	0.005	1.49	0.00001
	PI38:5	0.748	0.016	0.529	0.024	1.42	0.00001
	PI38:4	1.024	0.035	0.840	0.029	1.22	0.00019
	PI38:3	0.549	0.012	0.649	0.029	0.85	0.00076
	PI38:2	0.188	0.006	0.239	0.015	0.79	0.00074
	PI38:1	0.076	0.002	0.114	0.004	0.66	0.00000
	PI40:6	0.045	0.004	0.030	0.002	1.49	0.00087
	PI40:5	0.089	0.004	0.068	0.004	1.30	0.00030
	PI40:4	0.031	0.001	0.030	0.003	1.04	0.53164
	PI40:3	0.006	0.001	0.007	0.002	0.90	0.44126
	Sum	18.666		19.855		0.94	0.03907
PEP	PEP32:2	0.121	0.006	0.117	0.003	1.04	0.27399
	PEP32:1	0.271	0.013	0.280	0.012	0.97	0.37008
	PEP33:2	0.236	0.013	0.298	0.003	0.79	0.00009
	PEP33:1	0.388	0.003	0.518	0.012	0.75	0.00000
	PEP34:3	0.279	0.008	0.289	0.009	0.97	0.18624
	PEP34:2	0.971	0.013	0.913	0.023	1.06	0.00514
	PEP34:1	1.408	0.038	1.286	0.030	1.09	0.00242

	PEP35:2	0.213	0.007	0.170	0.005	1.25	0.00005
	PEP35:1	0.204	0.005	0.173	0.005	1.18	0.00018
	PEP36:5	0.285	0.007	0.133	0.003	2.15	0.00000
	PEP36:4	0.553	0.015	0.273	0.006	2.03	0.00000
	PEP36:3	0.638	0.025	0.447	0.015	1.43	0.00001
	PEP36:2	1.155	0.025	0.832	0.030	1.39	0.00000
	PEP36:1	0.747	0.013	0.612	0.009	1.22	0.00000
	PEP38:5	0.661	0.012	0.236	0.002	2.80	0.00000
	PEP38:4	0.379	0.009	0.168	0.006	2.26	0.00000
	PEP40:6	0.101	0.012	0.001	0.002	87.91	0.00000
	PEP40:5	0.231	0.004	0.069	0.002	3.32	0.00000
	Sum	8.842		6.814		1.30	0.00000
PE	PE28:0	0.071	0.002	0.230	0.006	0.31	0.00000
	PE29:0	0.012	0.000	0.044	0.001	0.27	0.00000
	PE30:1	0.161	0.006	0.377	0.003	0.43	0.00000
	PE30:0	0.140	0.004	0.441	0.015	0.32	0.00000
	PE31:0	0.034	0.001	0.120	0.005	0.28	0.00000
	PE32:2	0.226	0.001	0.471	0.013	0.48	0.00000
	PE32:1	0.754	0.013	1.843	0.047	0.41	0.00000
	PE32:0	0.210	0.003	0.463	0.016	0.45	0.00000
	PE33:1	0.236	0.004	0.541	0.021	0.44	0.00000
	PE33:0	0.054	0.002	0.126	0.008	0.43	0.00000
	PE34:3	0.577	0.010	0.859	0.020	0.67	0.00000
	PE34:2	3.413	0.046	5.037	0.126	0.68	0.00000
	PE34:1	4.523	0.063	6.966	0.228	0.65	0.00000
	PE35:2	0.640	0.010	0.745	0.018	0.86	0.00005
	PE35:1	0.611	0.004	0.792	0.030	0.77	0.00002
	PE36:5	0.438	0.014	0.496	0.018	0.88	0.00228
	PE36:4	2.722	0.048	2.917	0.072	0.93	0.00421
	PE36:3	10.111	0.092	11.136	0.160	0.91	0.00003
	PE36:2	23.077	0.223	27.481	0.594	0.84	0.00001
	PE36:1	7.676	0.071	8.984	0.224	0.85	0.00003
	PE37:3	0.102	0.001	0.115	0.006	0.89	0.00699
	PE37:2	0.205	0.004	0.272	0.008	0.75	0.00000
	PE38:6	0.592	0.016	0.498	0.009	1.19	0.00004
	PE38:5	1.790	0.027	1.713	0.034	1.04	0.01217
	PE38:4	1.303	0.023	1.296	0.024	1.01	0.67839
	PE38:3	0.441	0.011	0.532	0.008	0.83	0.00001
	PE38:2	0.232	0.009	0.315	0.007	0.74	0.00001
	PE38:1	0.113	0.001	0.169	0.003	0.67	0.00000
	PE40:6	0.681	0.057	0.524	0.037	1.30	0.00355
	PE40:5	0.698	0.007	0.595	0.011	1.17	0.00000
	Sum	61.844		76.097		0.81	0.00000
PS	PS 34:2	0.342	0.014	0.423	0.016	0.81	0.00026

	PS 34:1	1.051	0.019	1.276	0.036	0.82	0.00003
	PS 35:1	0.329	0.029	0.435	0.023	0.76	0.00124
	PS 36:3	2.286	0.038	2.238	0.066	1.02	0.25436
	PS 36:2	9.111	0.198	9.050	0.202	1.01	0.68177
	PS 36:1	12.280	0.174	12.733	0.351	0.96	0.06003
	PS 38:5	0.510	0.010	0.470	0.011	1.08	0.00173
	PS 38:4	0.873	0.012	0.880	0.017	0.99	0.50142
	PS 38:3	0.799	0.017	0.892	0.022	0.90	0.00057
	PS 38:2	0.537	0.013	0.540	0.007	0.99	0.71286
	PS 38:1	0.621	0.009	0.629	0.016	0.99	0.41291
	PS 40:6	1.052	0.011	0.614	0.019	1.71	0.00000
	PS 40:5	3.008	0.044	2.088	0.030	1.44	0.00000
	PS 40:3	0.087	0.006	0.100	0.004	0.86	0.00724
	PS 40:2	0.255	0.006	0.324	0.007	0.79	0.00001
	PS 40:1	0.372	0.012	0.514	0.018	0.72	0.00001
	Sum	33.511		33.207		1.01	0.55311
PCP	PCP30:0	0.091	0.003	0.123	0.004	0.75	0.00001
	PCP32:2	0.051	0.002	0.043	0.004	1.19	0.00710
	PCP32:1	0.183	0.002	0.183	0.005	1.00	0.92630
	PCP33:3	0.024	0.003	0.020	0.003	1.22	0.08696
	PCP33:2	0.148	0.006	0.150	0.001	0.99	0.55632
	PCP33:1	0.372	0.003	0.398	0.006	0.94	0.00018
	PCP34:3	0.063	0.005	0.059	0.005	1.07	0.30194
	PCP34:2	0.263	0.003	0.231	0.008	1.14	0.00037
	PCP34:1	0.491	0.009	0.379	0.009	1.29	0.00000
	PCP34:0	0.196	0.005	0.020	0.006	9.82	0.00000
	PCP35:1	0.039	0.005	0.018	0.005	2.15	0.00097
	PCP36:4	0.052	0.002	0.024	0.002	2.16	0.00000
	PCP36:3	0.091	0.003	0.042	0.003	2.16	0.00000
	PCP36:2	0.166	0.004	0.088	0.003	1.89	0.00000
	PCP36:1	0.208	0.006	0.123	0.004	1.68	0.00000
	PCP37:5	0.021	0.002	0.009	0.001	2.46	0.00007
	PCP38:5	0.038	0.002	0.010	0.004	3.76	0.00002
	PCP38:4	0.033	0.005	0.009	0.001	3.81	0.00013
	Sum	2.532		1.929		1.31	0.00000
PC	PC28:0	1.528	0.046	2.539	0.036	0.60	0.00000
	PC29:0	0.403	0.010	0.790	0.011	0.51	0.00000
	PC30:1	0.431	0.014	0.628	0.014	0.69	0.00000
	PC30:0	6.140	0.142	10.053	0.148	0.61	0.00000
	PC31:1	0.139	0.003	0.232	0.006	0.60	0.00000
	PC31:0	1.344	0.021	2.349	0.050	0.57	0.00000
	PC32:2	0.541	0.007	0.698	0.006	0.78	0.00000
	PC32:1	3.571	0.064	4.781	0.048	0.75	0.00000
	PC32:0	7.353	0.124	8.977	0.080	0.82	0.00000

	PC33:3	0.040	0.002	0.061	0.002	0.66	0.00001
	PC33:2	0.206	0.003	0.295	0.004	0.70	0.00000
	PC33:1	1.216	0.017	1.697	0.020	0.72	0.00000
	PC33:0	0.972	0.013	1.320	0.022	0.74	0.00000
	PC34:3	1.052	0.011	1.079	0.018	0.97	0.04000
	PC34:2	4.981	0.045	5.403	0.065	0.92	0.00004
	PC34:1	17.276	0.108	18.108	0.198	0.95	0.00032
	PC34:0	3.756	0.068	3.501	0.320	1.07	0.16972
	PC35:3	0.144	0.003	0.142	0.003	1.01	0.44774
	PC35:2	0.648	0.008	0.597	0.007	1.09	0.00008
	PC35:1	1.235	0.014	1.235	0.013	1.00	0.99521
	PC35:0	0.302	0.006	0.332	0.005	0.91	0.00024
	PC36:5	0.430	0.002	0.363	0.003	1.19	0.00000
	PC36:4	2.108	0.019	1.661	0.022	1.27	0.00000
	PC36:3	5.986	0.047	4.739	0.044	1.26	0.00000
	PC36:2	15.669	0.087	11.791	0.105	1.33	0.00000
	PC36:1	7.938	0.062	6.304	0.026	1.26	0.00000
	PC36:0	0.131	0.034	0.117	0.026	1.12	0.52891
	PC37:3	0.064	0.002	0.054	0.002	1.17	0.00054
	PC37:2	0.157	0.007	0.129	0.004	1.22	0.00041
	PC37:1	0.101	0.004	0.098	0.003	1.02	0.39788
	PC37:0	0.013	0.002	0.019	0.001	0.67	0.00084
	PC38:6	0.362	0.007	0.242	0.007	1.50	0.00000
	PC38:5	0.988	0.018	0.741	0.015	1.33	0.00000
	PC38:4	0.625	0.009	0.507	0.009	1.23	0.00000
	PC38:3	0.315	0.007	0.285	0.008	1.11	0.00133
	PC38:2	0.356	0.006	0.348	0.006	1.02	0.08217
	PC38:1	0.267	0.004	0.315	0.005	0.85	0.00001
	PC40:7	0.134	0.009	0.062	0.003	2.15	0.00001
	PC40:6	0.283	0.012	0.160	0.001	1.77	0.00000
	PC40:5	0.232	0.006	0.139	0.004	1.67	0.00000
	PC40:4	0.063	0.008	0.044	0.003	1.42	0.00452
	PC40:3	0.017	0.002	0.023	0.002	0.77	0.00575
	Sum	89.520		92.959		0.96	0.00013
SM	SM32:1	1.282	0.049	1.326	0.031	0.97	0.18265
	SM32:0	0.527	0.023	0.894	0.024	0.59	0.00000
	SM33:2	0.026	0.001	0.029	0.001	0.90	0.00456
	SM33:1	1.133	0.034	1.205	0.029	0.94	0.01751
	SM34:2	0.355	0.013	0.272	0.010	1.31	0.00006
	SM34:1	9.852	0.272	8.148	0.142	1.21	0.00003
	SM35:2	0.069	0.005	0.053	0.004	1.31	0.00207
	SM35:1	1.024	0.030	0.742	0.012	1.38	0.00000
	SM36:2	0.411	0.013	0.197	0.003	2.09	0.00000
	SM36:1	2.824	0.092	1.226	0.026	2.30	0.00000

	SM37:1	0.766	0.038	0.447	0.008	1.72	0.00000
	SM38:2	0.271	0.018	0.138	0.007	1.96	0.00001
	SM38:1	6.150	0.297	4.913	0.137	1.25	0.00028
	SM39:2	1.291	0.053	1.561	0.037	0.83	0.00016
	SM39:1	6.315	0.249	7.589	0.193	0.83	0.00019
	SM40:2	2.728	0.122	2.108	0.047	1.29	0.00008
	SM40:1	12.402	0.513	10.966	0.287	1.13	0.00275
	SM41:2	3.270	0.138	3.250	0.088	1.01	0.82009
	SM41:1	7.821	0.377	8.369	0.211	0.93	0.04435
	SM42:2	3.295	0.137	2.253	0.060	1.46	0.00001
	SM42:1	6.273	0.299	5.366	0.155	1.17	0.00169
	SM43:2	1.196	0.061	0.904	0.018	1.32	0.00010
	SM43:1	1.070	0.067	0.888	0.023	1.21	0.00219
	Sum	70.351		62.842		1.12	0.00335
LPC	LPC15:0	0.037	0.002	0.063	0.002	0.58	0.00000
	LPC16:0	0.595	0.019	0.712	0.006	0.84	0.00002
	LPC16:1	0.031	0.002	0.043	0.002	0.71	0.00008
	LPC17:0	0.026	0.001	0.032	0.001	0.79	0.00005
	LPC18:3	0.153	0.004	0.234	0.001	0.66	0.00000
	LPC18:2	0.147	0.004	0.195	0.007	0.75	0.00002
	LPC18:1	0.588	0.011	0.742	0.014	0.79	0.00000
	LPC18:0	0.170	0.006	0.194	0.007	0.88	0.00173
	sum	1.747		2.215		0.79	0.00000
AcylCar	CAR 2:0	17.362	0.354	9.257	0.271	1.88	0.00000
	CAR 3:0	4.101	0.134	1.650	0.041	2.49	0.00000
	CAR 4:0	4.724	0.053	1.102	0.010	4.29	0.00000
	CAR 5:0	3.573	0.106	1.448	0.016	2.47	0.00000
	CAR 6:0	0.027	0.001	0.013	0.000	2.11	0.00000
	Sum	29.786		13.469		2.21	0.00000
LacCer	LacCer 32:1	0.028	0.001	0.072	0.002	0.38	0.00000
	LacCer 34:1	0.348	0.012	0.622	0.011	0.56	0.00000
	LacCer 36:1	0.123	0.003	0.109	0.005	1.13	0.00207
	LacCer 38:1	0.264	0.008	0.436	0.015	0.60	0.00000
	LacCer 39:1	0.243	0.012	0.618	0.017	0.39	0.00000
	LacCer 40:1	0.724	0.032	1.295	0.029	0.56	0.00000
	LacCer 41:1	0.487	0.016	1.134	0.031	0.43	0.00000
	LacCer 42:1	0.420	0.019	0.733	0.026	0.57	0.00000
	Sum	2.635		5.020		0.53	0.00000
TAG	TAG26:0	92.545	1.146	88.704	0.558	1.04	0.00643
	TAG28:1	44.420	0.909	33.894	0.656	1.31	0.00008
	TAG28:0	183.817	2.263	203.630	3.551	0.90	0.00123
	TAG29:0	23.050	0.414	31.102	0.534	0.74	0.00003
	TAG30:1	88.768	1.534	76.172	1.962	1.17	0.00094
	TAG30:0	242.308	2.607	299.342	3.184	0.81	0.00002

TAG31:0	35.134	0.534	55.224	1.183	0.64	0.00001
TAG32:2	30.433	0.406	25.338	0.614	1.20	0.00028
TAG32:1	154.095	2.786	142.215	2.305	1.08	0.00471
TAG32:0	348.326	3.448	469.456	9.431	0.74	0.00003
TAG33:0	65.640	0.750	117.257	1.721	0.56	0.00000
TAG34:2	43.748	0.506	41.446	0.979	1.06	0.02239
TAG34:1	241.104	3.893	249.648	4.414	0.97	0.06573
TAG34:0	531.462	0.428	741.695	8.347	0.72	0.00000
TAG35:0	121.871	0.723	214.643	3.267	0.57	0.00000
TAG36:2	108.964	0.797	115.229	2.427	0.95	0.01318
TAG36:1	481.378	10.139	487.375	2.883	0.99	0.38015
TAG36:0	640.081	7.241	909.003	10.342	0.70	0.00000
TAG37:1	107.543	0.879	120.331	2.453	0.89	0.00105
TAG37:0	141.342	1.152	214.340	5.152	0.66	0.00002
TAG38:2	272.124	1.970	241.204	5.075	1.13	0.00060
TAG38:1	751.681	12.767	715.938	11.382	1.05	0.02237
TAG38:0	589.633	4.269	777.152	11.487	0.76	0.00001
TAG39:1	117.159	1.332	116.436	3.295	1.01	0.74224
TAG39:0	95.254	1.264	140.000	1.549	0.68	0.00000
TAG40:3	190.088	1.745	128.295	2.633	1.48	0.00000
TAG40:2	459.302	2.991	324.879	3.728	1.41	0.00000
TAG40:1	622.856	5.777	561.733	8.898	1.11	0.00057
TAG40:0	392.862	2.377	521.051	11.541	0.75	0.00005
TAG41:1	57.051	0.883	68.237	1.370	0.84	0.00029
TAG41:0	56.391	0.615	105.130	0.966	0.54	0.00000
TAG42:3	78.023	0.718	63.224	1.503	1.23	0.00010
TAG42:2	202.830	2.745	171.139	1.241	1.19	0.00005
TAG42:1	339.919	3.454	374.468	7.686	0.91	0.00208
TAG42:0	198.518	1.495	293.605	6.520	0.68	0.00002
TAG43:1	44.262	1.102	64.899	0.831	0.68	0.00001
TAG43:0	41.169	0.541	84.144	1.946	0.49	0.00000
TAG44:3	42.953	0.561	44.175	0.518	0.97	0.05039
TAG44:2	134.816	1.428	149.625	2.081	0.90	0.00053
TAG44:1	222.541	2.372	267.898	5.277	0.83	0.00017
TAG44:0	123.193	2.547	195.805	1.554	0.63	0.00000
TAG45:2	17.271	0.298	20.897	0.581	0.83	0.00065
TAG45:1	43.825	0.903	67.466	2.005	0.65	0.00005
TAG45:0	31.111	0.678	66.760	1.153	0.47	0.00000
TAG46:3	55.553	0.769	59.887	0.917	0.93	0.00330
TAG46:2	141.878	1.178	152.371	3.275	0.93	0.00642
TAG46:1	172.391	2.014	207.672	2.637	0.83	0.00005
TAG46:0	77.199	1.179	131.433	1.621	0.59	0.00000
TAG47:2	23.207	0.248	30.599	0.721	0.76	0.00007
TAG47:1	45.842	0.269	73.542	1.248	0.62	0.00000

TAG47:0	21.890	0.313	50.732	0.196	0.43	0.00000
TAG48:3	52.654	0.825	63.536	1.519	0.83	0.00040
TAG48:2	128.329	1.163	150.090	1.234	0.86	0.00002
TAG48:1	151.940	2.678	192.086	3.636	0.79	0.00010
TAG48:0	51.539	0.510	91.472	0.995	0.56	0.00000
TAG49:2	34.092	0.563	43.870	0.636	0.78	0.00004
TAG49:1	54.398	0.556	84.901	0.811	0.64	0.00000
TAG49:0	23.935	0.949	45.146	1.314	0.53	0.00002
TAG50:4	30.930	0.589	35.489	0.836	0.87	0.00152
TAG50:3	84.833	0.789	92.201	1.732	0.92	0.00257
TAG50:2	149.541	1.229	154.434	2.523	0.97	0.03918
TAG50:1	142.016	2.651	181.650	2.184	0.78	0.00004
TAG50:0	55.520	0.640	85.974	0.662	0.65	0.00000
TAG51:3	21.380	0.432	21.908	0.430	0.98	0.20855
TAG51:2	51.261	0.405	57.468	1.393	0.89	0.00177
TAG51:1	54.947	0.178	71.530	1.903	0.77	0.00011
TAG51:0	23.881	0.590	35.952	0.393	0.66	0.00001
TAG52:4	55.942	0.411	51.277	1.223	1.09	0.00332
TAG52:3	110.914	1.028	95.394	0.549	1.16	0.00002
TAG52:2	169.429	2.930	148.256	1.789	1.14	0.00044
TAG52:1	136.417	1.492	138.126	1.125	0.99	0.18828
TAG52:0	55.847	0.298	63.278	1.273	0.88	0.00060
TAG53:2	37.180	0.148	34.170	0.740	1.09	0.00230
TAG53:1	33.807	1.066	36.529	0.646	0.93	0.01942
TAG54:5	43.872	0.382	29.572	0.621	1.48	0.00000
TAG54:4	74.510	1.064	48.303	0.564	1.54	0.00000
TAG54:3	103.186	1.589	66.835	0.899	1.54	0.00000
TAG54:2	100.909	0.521	72.454	0.829	1.39	0.00000
TAG54:1	70.834	0.527	59.474	1.007	1.19	0.00007
TAG55:2	8.805	0.237	9.557	0.044	0.92	0.00569
TAG55:1	11.049	0.155	14.856	0.139	0.74	0.00001
Sum	11512.715		13182.227		0.87	0.00018

FC (fold change): NEB / PEB. Values are means of 4 measurements of pooled samples.
